# The combined effect of socioeconomic position and C-reactive protein for predicting incident cardiometabolic disease: Findings from a 14-year follow-up study of the English Longitudinal Study of Ageing (ELSA)

**DOI:** 10.1016/j.ssmph.2023.101520

**Published:** 2023-09-25

**Authors:** Lydia Poole, Antonio I. Lazzarino, Kimberley J. Smith, Ruth A. Hackett

**Affiliations:** a Department of Psychological Interventions, School of Psychology, University of Surrey, Guildford, United Kingdom; b Department of Epidemiology and Biostatistics, Imperial College London, London, United Kingdom; c Health Psychology Section, Department of Psychology, Institute of Psychiatry, Psychology and Neuroscience, King's College London, London, United Kingdom

**Keywords:** Socioeconomic position, Wealth, C-reactive protein, Coronary heart disease, Stroke, Diabetes, Multimorbidity, Longitudinal

## Abstract

Cardiovascular disease and diabetes are leading causes of morbidity and mortality worldwide. Social inequalities in the distribution of these diseases across the population exist. The aim of the current study was to examine the additive effect of socioeconomic position and a known biological risk marker (C-reactive protein [CRP]) for future incident cardiometabolic disease. We used data from the English Longitudinal Study of Ageing (N = 5410). Tertiles of net financial wealth and CRP (>3 mg/L) were measured at wave 2 (2004/05) and disease incidence (coronary heart disease [CHD], stroke, diabetes/high blood glucose) was reported across the subsequent 14 years of follow-up (2006–2019). Individual diseases were modelled as well as cardiometabolic multimorbidity which was defined as 2 or more incident cardiometabolic disease diagnoses over follow-up. Participants were free from the disease of interest at baseline. Cox proportional hazard and logistic regression analyses were used controlling for sociodemographic, lifestyle and health-related covariates. After adjusting for all covariates, the combination of low wealth and elevated CRP was an independent predictor of incident diabetes/high blood glucose (Hazard Ratio (HR) = 2.14; 95% Confidence Interval (C.I.) = 1.49–3.07), CHD (HR = 2.48, 95% C.I. = 1.63–3.76), stroke (HR = 1.55; 95% C.I. = 1.18–2.04), relative to high wealth/low CRP. Low wealth and elevated CRP was also an independent predictor of incident cardiometabolic multimorbidity (Odds Ratio = 2.22, 95% C.I. = 1.16–4.28) in age and sex adjusted models. The presence of both low wealth and elevated CRP was implicated in the onset of CHD, stroke, diabetes/high blood glucose, and cardiometabolic multimorbidity up to 14 years later, reflecting the role of psychobiological processes in predicting disease burden. Our results reinforce calls for efforts to tackle structural inequalities to improve healthy ageing trajectories.

## Highlights

• Socioeconomic factors are known to adversely affect future health.
• Biological factors such as inflammation also predict future disease.
• The combination of both factors for future cardiometabolic disease was explored.
• Low wealth plus elevated C-reactive protein predicted incident diseases.
• Findings reflect the detrimental effect of multiple risk factors for healthy ageing processes.

## 1 Introduction

Cardiovascular disease and diabetes are leading causes of morbidity and mortality worldwide (Sattar et al., 2020). In the United Kingdom (UK), socioeconomic inequalities in health are well recognised and are stark (Marmot, 2020); disparities extend to long-term conditions including cardiovascular disease and diabetes. Indeed, several markers of socioeconomic position (SEP) have been associated with poor cardiovascular outcomes, including educational attainment, income level, environmental factors and employment status (Schultz et al., 2018), with low SEP associated with greater risk of both cardiovascular related risk factors (e.g. hypertension) as well as incident disease (de Mestral & Stringhini, 2017). Similar evidence exists for the link between SEP and diabetes (Hill-Briggs et al., 2020). For example, a recent large multi-cohort study of over 100,000 participants linked low SEP with new onset diabetes (Kivimäki et al., 2020). However, despite cardiovascular disease and diabetes often presenting as co-morbid conditions (Einarson et al., 2018), little evidence exists for the association between SEP and cardiometabolic multimorbidity specifically.

Other factors are also known to increase risk of cardiometabolic diseases, some of which are also known to intersect with SEP. These include the role of adverse health behaviours (Mackenbach et al., 2019), discrimination (Churchwell et al., 2020; Jackson et al., 2019), and structural (e.g. unable to take time off work) and individual (e.g. low health literacy) level barriers to accessing healthcare (Diederichs et al., 2018; Schultz et al., 2018). SEP is also known to be associated with biomarkers of future disease. In 2020, Muscatel and colleagues conducted a meta-analysis of studies examining the association between SEP and IL-6 and CRP, finding individuals with lower SEP showed significantly higher levels of systemic inflammation than those with higher SEP (Muscatell et al., 2020). Cardiovascular disease and diabetes both involve inflammatory processes; indeed, inflammation has been associated with diabetes incidence (Bellou et al., 2018) and poor prognosis and mortality in cardiovascular disease (Ni et al., 2020). However, the extent to which inflammatory processes and SEP exert a combined effect leading to increased risk of future incident cardiometabolic disease over time is less clear.

The aim of this study was to examine the longitudinal association between wealth and CRP for predicting incident cardiometabolic disease (coronary heart disease [CHD], stroke, diabetes/high blood glucose) using data from a nationally representative cohort study of English middle aged and older adults. In particular, we were interested in examining the additive effect of wealth in combination with CRP on future disease. We selected wealth as a measure of SEP since in older adults it provides a more proximal measure of disadvantage than other possible measures such as education and employment status (Banks et al., 2008; Robert & House, 1996). Secondary objectives were to model the onset of cardiometabolic multimorbidity over time (2 or more incident disease diagnoses over follow-up) (The Academy of Medical Sciences, 2018).

## 2 Methods

### 2.1 Participants and study design

**Fig. 1:**
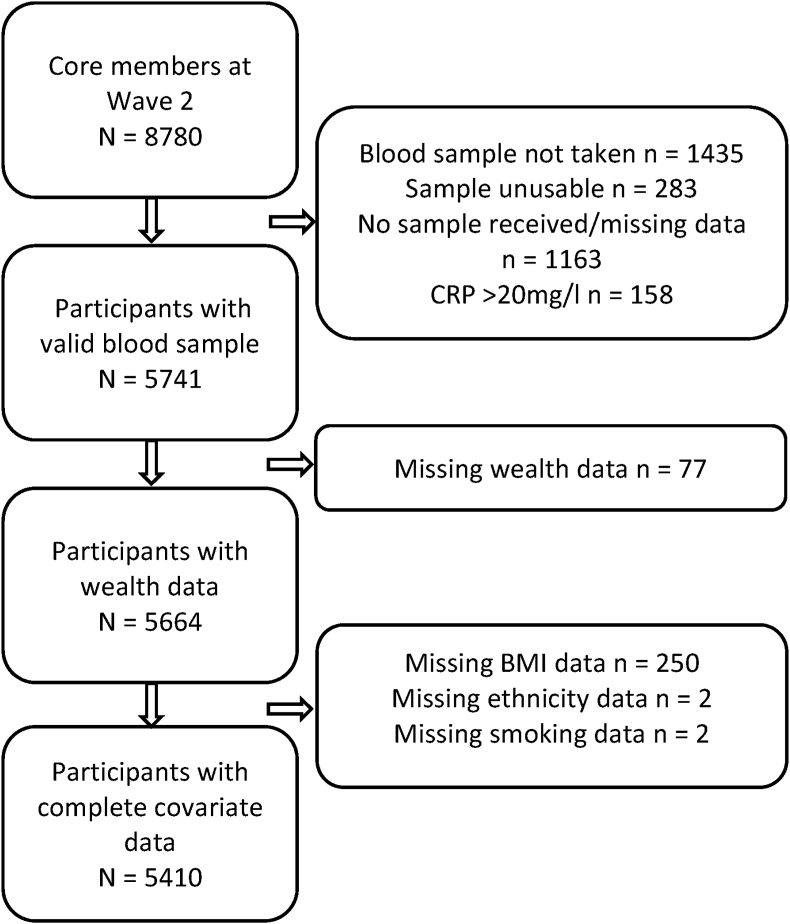
Flow diagram of analytic sample size. BMI = Body Mass Index; CRP=C-reactive protein.

Our sample was drawn from the English Longitudinal Study of Ageing (ELSA), a nationally representative general population study of adults aged 50 years and older living in England (Steptoe et al., 2012). The sample is followed up biennially; at all waves participants complete a computer-assisted personal interview plus a self-completion questionnaire. On alternate waves, a nurse visit enables the collection of blood samples and objective assessments of physical function. This current paper uses data collected across 14 years of study, from wave 2 (2004/5) through to wave 9 (2018/2019). Out of a total possible sample of 8780 core members participating at wave 2, 5410 were included in the analyses presented here, having complete data on all independent variables and covariates. Fig. 1 illustrates how the analytic sample was derived based on complete data. Compared to those included, those excluded in the analytic sample were more likely to be female (χ^2^ (df = 1) = 5.62, *p* = 0.018), non-smoking (χ^2^ (df = 1) = 21.25, *p* < 0.001), White (χ^2^ (df = 1) = 36.92, *p* < 0.001), of older age *t* (df = 8778) = 13.89, *p* < 0.001), and higher body mass index (BMI; *t* (df = 7223) = 5.38, *p* < 0.001); they were also more likely to be cohabiting (χ^2^ (df = 1) = 72.05, *p* < 0.001). Since different models were performed to predict each incident cardiometabolic disease, excluding those reporting the respective disease at any point up to and including the baseline assessment, specific Ns are reported for the individual models.

### 2.2 Measures

#### 2.2.1 Predictor variables: wealth and C-reactive protein

Wave 2 SEP was included in models as tertiles of net financial wealth, which refers to participants’ gross financial wealth with financial debt subtracted. Wealth is the most robust measure of SEP in ELSA, and has been found to be more strongly associated with the risk of death than any other SEP measure in this cohort (Demakakos et al., 2016).

High sensitivity CRP was assessed from blood drawn by study nurses at wave 2. Participants who had a clotting or bleeding disorder and those on anti-coagulant medication did not provide blood samples. CRP was measured using the N Latex CRP mono immunoassay on the Behring Nephelometer II analyser. All blood samples were analysed at the Royal Victoria Infirmary laboratory in Newcastle upon Tyne, UK (for a detailed description of blood analyses see (Sproston & Mindell, 2004). Outliers above 20 mg/L (n = 158) were removed from analyses since these may indicate the presence of an acute infection (Holm et al., 2007). A binary variable using a cut-off of >3 mg/L was used to indicate those with low and high values (He et al., 2010).

#### 2.2.2 Outcome variables: incident cardiometabolic disease

Participants were shown a list of illnesses and asked to self-report any doctor-diagnosed illnesses they had developed since the previous ELSA wave. Incident cardiometabolic disease was defined as a new positive report of CHD, stroke, or diabetes/high blood glucose by participants at waves 3, 4, 5, 6, 7, 8 and 9, excluding participants who reported that same disease at wave 2 baseline. For the purposes of our analyses, CHD was defined to include all cases of angina and myocardial infarction. Cardiometabolic multimorbidity was computed as a reporting of two or more incident illnesses (CHD, stroke, diabetes/high blood glucose) over follow-up (The Academy of Medical Sciences, 2018).

#### 2.2.3 Covariates

Covariates were all measured at baseline (wave 2) and were selected for inclusion in models based on their known relationship with either the exposure or outcome variables. Sociodemographic variables included in models were age (measured in years), sex (male/female), and whether participants were cohabiting with a partner (cohabiting/non-cohabiting). Ethnicity was coded as a binary variable (White/ethnic minority). Height and weight were collected during the nurse visit and BMI was derived using the standard formula (kg/m^2^). Whether or not participants reported being a current smoker (no/yes) was also included. Participants reported the frequency in which they engaged in vigorous, moderate and mild physical activity and we used this data to derive three possible categories reflecting regularity of physical activity: none/mild activity only per week, moderate/vigorous activity once a week, moderate/vigorous activity more than once a week. Doctor diagnosis of hypertension or use of anti-hypertensive medication was self-reported, and these responses were combined with objective assessments taken at the nurse visit (hypertension defined as systolic blood pressure >140 mmHg and diastolic blood pressure >90 mmHg) to generate a binary variable (no/yes). In sensitivity analyses, glycated haemoglobin (HbA1c; %) and total cholesterol (mmol/l) were included as additional covariates in models. HbA1c and total cholesterol was assessed from blood drawn from participants' forearms. Participants who had a clotting or bleeding disorder and those on anti-coagulant medication did not provide blood samples. Fasting samples, defined as not eating or drinking for 5 h prior to blood draw, were collected where possible and when not otherwise contraindicated (e.g., >80 years, known diabetes, frail or unwell, ever had a seizure). In other sensitivity analyses we additionally included wave 2 depression symptom scores as measured on the Centre for Epidemiological Studies Depression 8-item scale (CES-D) (Radloff, 1977; Steffick, 2000), based on our previous work showing this to be an important risk factor for future disease in ELSA (Poole & Steptoe, 2018). We computed a summary score by adding responses to all eight dichotomous questions (possible range: 0–8). The Cronbach's alpha for the CES-D in this study was 0.782.

### 2.3 Statistical analysis

The effect of wealth and CRP was calculated by combining the two variables into a categorical variable comprising high wealth/low CRP (reference group), high wealth/high CRP, intermediate wealth/low CRP, intermediate wealth/high CRP, low wealth/low CRP and low wealth/high CRP. Chi-square analyses were used to assess the association between wealth and CRP. First, we performed analyses to examine the association between wealth/CRP at baseline on individual incident diseases over follow-up. Using adjusted Cox proportional hazard regression analyses, participants with each specific illness were excluded from the sample at baseline; therefore, separate Ns are reported for these models. Including variables that could plausibly fall on the causal pathway between the exposure and outcome can introduce bias into effect estimates (Wang et al., 2017); hence, we present minimally adjusted models controlling for age and sex only, as well as fully adjusted models. Results for all models are presented as adjusted hazard ratios (HR) with 95% confidence intervals (C.I.). Fully adjusted models included the covariates: age, sex, cohabitation status, ethnicity, smoking status, BMI, physical activity, and hypertension status.

To test for interaction on the additive scale we calculated the relative excess risk due to interaction (RERI) for each disease outcome using the minimally adjusted effect estimates. RERI was calculated following the guidelines of Knol et al. (2011), using the supplementary file provided by Knol and VanderWeele (2012) to calculate 95% C.I. An additive interaction effect indicates that the presence of two factors (e.g., wealth and CRP) is needed to cause an outcome (e.g., cardiometabolic disease). RERI >0 indicates that having both factors (e.g., low wealth and elevated CRP) surpasses the sum of the individual effects of these exposures (Knol et al., 2011).

The supplementary file displays the results of sensitivity analyses. The first sensitivity analysis included HbA1c in models to predict incident illness. The second sensitivity analysis included total cholesterol in models to predict incident illness. The third sensitivity analysis removed all cases (those with and without incident disease) within the two years following baseline, as a check for reverse causation. The final sensitivity analysis included CES-D to models to predict each outcome in turn (diabetes/high blood glucose, CHD and stroke). The assumption of proportional hazards was assessed by visually inspecting log minus log plots for each of the outcomes; no intersecting lines were observed for wealth/CRP and therefore the assumption was deemed to be upheld.

We performed additional secondary analyses. First, we used logistic regression to examine the association between wealth/CRP at baseline on incident cardiometabolic multimorbidity (a binary outcome); results are presented as adjusted odds ratios (OR) with 95% C.I. Second, we tested the multiplicative interaction using mean centred continuous wealth*mean centred continuous CRP to model whether CRP moderated the association between wealth and incident cardiometabolic diseases using fully adjusted Cox regression.

All analyses were conducted using SPSS version 24. Two-tailed tests were used throughout, and the significance level was set at p < 0.05, though exact significance levels are reported.

## 3 Results

Table 1 shows the characteristics of the sample. The average participant was 65.80 years old (±9.42 years) and married or cohabiting. A minority of participants were current smokers (13.8%). The average BMI was above 25 kg/m^2^, reflective of the high levels of obesity in the sample (BMI >30 kg/m^2^ = 27.4%). Mean net financial wealth was £61,281.95 (±£162,162.90). A third of participants had heightened levels of CRP (>3 mg/l). Chi-square analyses revealed a significant association between wealth tertile and CRP (χ^2^ (df = 2) = 108.99, *p* < 0.001), with 41.0% of those in the lowest wealth tertile, 33.3% of those in the intermediate wealth tertile, and 24.6% of those in the highest wealth tertile, having elevated CRP >3 mg/l.

**Table 1:** Demographic, clinical and biological characteristics of the sample (N = 5410).

Characteristic	Mean ± SD or N(%)
**Baseline sociodemographics**	
Age (years)	65.80 ± 9.42
Female	2923 (54.0%)
White ethnicity	5327 (98.5%)
Married/cohabiting	3850 (71.2%)
Net financial wealth – tertiles	
1 (highest)	1995 (36.9%)
2	1852 (34.2%)
3 (lowest)	1563 (28.9%)
**Baseline health variables**	
Current smoker	744 (13.8%)
Body mass index (kg/m^2^)	27.75 ± 4.71
Physical activity	
None/only light	1032 (19.1%)
Moderate/vigorous sessions ≤1 a week	1276 (23.6%)
Moderate/vigorous sessions >1 a week	3102 (57.3%)
Hypertension	3073 (56.8%)
HbA1c % (*n* = 5293)	5.59 ± 0.73
Total cholesterol mmol/l (*n* = 5409)	5.93 ± 1.20
CES-D (*n* = 5358)	1.43 ± 1.87
hs-CRP	3.07 ± 3.34
hs-CRP (>3 mg/l)	1749 (32.3%)
**Baseline wealth and CRP**	
Wealth/CRP combined	
High wealth/low CRP	1504 (27.8%)
High wealth/high CRP	491 (9.1%)
Intermediate wealth/low CRP	1235 (22.8%)
Intermediate wealth/high CRP	617 (11.4%)
Low wealth/low CRP	922 (17.0%)
Low wealth/high CRP	641 (11.9%)
**Baseline disease**	
Diabetes/high blood glucose	405 (7.5)
CHD	639 (11.8)
Stroke	183 (3.4)
**Incident disease at follow-up (waves 3 to 9)**	
Diabetes/high blood glucose (*n* = 4383)	416 (9.5%)
CHD (*n* = 4190)	290 (5.4%)
Stroke (*n* = 4583)	647 (12.0%)
Cardiometabolic multimorbidity (*n* = 3987)	101 (2.5%)

*CES-D = Centre for Epidemiological Studies Depression scale; CHD = coronary heart disease; CRP=C-reactive protein; HbA1c = glycated haemoglobin; hs = high sensitivity.

**Fig. 2:**
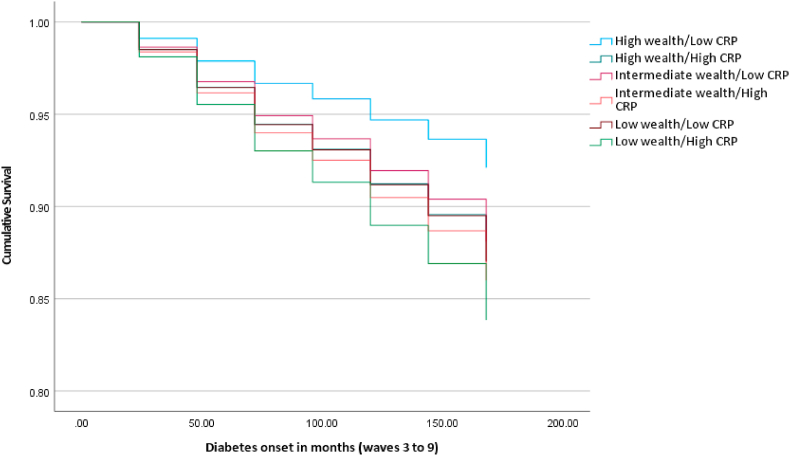
Survival plot for incident diabetes by wealth/CRP additive effect (N = 4383) Horizontal axis = time in months since baseline (2004/2005) Results are adjusted for age, sex, cohabitation, ethnicity, physical activity, smoking, body mass index, and hypertension CRP = C-reactive protein.

**Fig. 3:**
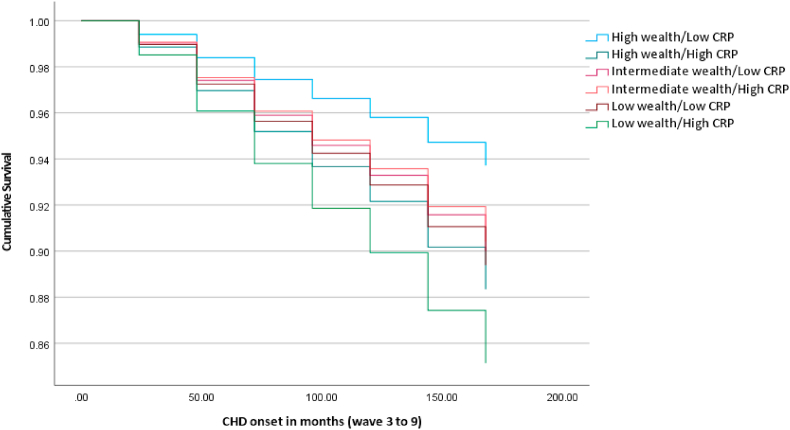
Survival plot for incident CHD by wealth/CRP additive effect (N = 4190) Horizontal axis = time in months since baseline (2004/2005) Results are adjusted for age, sex, cohabitation, ethnicity, smoking, physical activity, body mass index, and hypertension CHD = coronary heart disease; CRP=C-reactive protein.

**Fig. 4:**
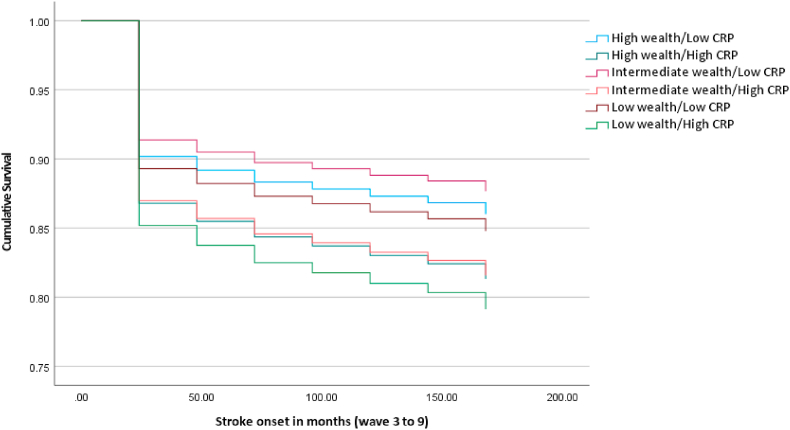
Survival plot for incident stroke by wealth/CRP additive effect (N = 4583) Horizontal axis = time in months since baseline (2004/2005) Results are adjusted for age, sex, cohabitation, ethnicity, smoking, physical activity, body mass index, and hypertension CRP = C-reactive protein.

Table 2 displays the results of the individual regression models performed for each incident cardiometabolic illnesses (Cox regression), and cardiometabolic multimorbidity (logistic regression). Models 1–3 (Table 2) reveal significant effects of the wealth/CRP groups for incident diabetes/high blood glucose, CHD and stroke. With regards to models predicting incident diabetes/high blood glucose and CHD, all combinations of wealth and CRP were associated with increased hazard of incident disease relative to those with high wealth and low CRP. Specifically, in fully adjusted models, participants with a combination of low wealth and elevated CRP were at over two times increased hazard of developing incident diabetes/high blood glucose (HR 2.14 95% C.I. 1.49; 3.07) and CHD (HR 2.48, 95% C.I. 1.63; 3.76) over the follow-up period relative to the high wealth and low CRP group. Specifically, crude estimates suggest that the proportion of individuals developing diabetes/high blood glucose was approximately 16.85% in the low wealth/elevated CRP group compared to 5.37% in the high wealth/low CRP reference group (see Table 2). Similar findings were observed for incident CHD (11.13% of low wealth/elevated CRP group vs. 4.47% in the high wealth/low CRP group). In sensitivity models, additional adjustment for baseline HbA1c and total cholesterol did not alter the pattern of findings (see Supplementary Tables 1 and 2). HbA1c was a significant predictor of diabetes/high blood glucose incidence (HR 2.27, 95% C.I. 2.11; 2.45) and total cholesterol significantly predicted increased hazard of CHD (HR 1.11, 95% C.I. 1.00; 1.22). The association with incident stroke appeared to be driven by elevated CRP, as opposed to differences in wealth (high wealth/high CRP: HR 1.37, 95% C.I. 1.04; 1.81). Using age and sex adjusted models, the RERI for the incidence of diabetes/high blood glucose was 0.68 (95% C.I. −1.20; 2.55) and for stroke was 0.18 (95% C.I. −0.47; 0.83), indicating non-significant super-additive effects; the RERI for CHD was −0.01 (95% C.I. −1.65; 1.64) suggesting no additive effect. Fig. 2, Fig. 3, Fig. 4 graphically display the survival curves for each of the three Cox regression models, illustrating that the fastest rate to disease onset similarly occurs in the low wealth/high CRP group. Sensitivity analyses removing cases with disease onset in the two years following baseline did not change the pattern of results (see Supplementary Table 3). Sensitivity analyses additionally controlling for depression scores (see Supplementary Table 4) did not change the results for the effect of wealth/CRP; CES-D scores were significantly associated with diabetes/high blood glucose and stroke (both *p* < 0.05) but not CHD.

**Table 2:** Prospective associations between baseline wealth/CRP additive interaction and incident individual and multimorbid cardiometabolic disease over 14 years.

Model	Incident disease outcome	N		Cases/N (total) (%)	Age and sex adjusted effect estimate (95% C.I.)	*p*	Fully adjusted effect estimate^a^ (95% C.I.)	*p*
1	Diabetes/high blood glucose	4383	↑wealth/↓CRP	68/1266 (5.37%)	Reference	–	Reference	–
			↑wealth/↑CRP	50/415 (12.05%)	2.46 (1.71–3.55)	**<0.001**	1.68 (1.16–2.44)	**0.006**
			↔wealth/↓CRP	85/1033 (8.23%)	1.70 (1.24–2.34)	**0.001**	1.54 (1.12–2.12)	**0.008**
			↔wealth/↑CRP	66/505 (13.07%)	3.06 (2.18–4.30)	**<0.001**	1.83 (1.29–2.61)	**<0.001**
			↓wealth/↓CRP	69/701 (9.84%)	2.31 (1.65–3.22)	**<0.001**	1.69 (1.20–2.39)	**0.003**
			↓wealth/↑CRP	78/463 (16.85%)	4.44 (3.20–6.17)	**<0.001**	2.14 (1.49–3.07)	**<0.001**
2	CHD	4190	↑wealth/↓CRP	55/1230 (4.47%)	Reference	–	Reference	–
			↑wealth/↑CRP	36/400 (9.00%)	2.09 (1.37–3.18)	**<0.001**	1.91 (1.24–2.93)	**0.003**
			↔wealth/↓CRP	70/984 (7.11%)	1.67 (1.18–2.38)	**0.004**	1.62 (1.14–2.32)	**0.008**
			↔wealth/↑CRP	34/482 (7.05%)	1.75 (1.14–2.69)	**0.011**	1.55 (1.00–2.41)	0.051
			↓wealth/↓CRP	46/654 (7.03%)	1.93 (1.30–2.85)	**0.001**	1.73 (1.15–2.59)	**0.008**
			↓wealth/↑CRP	49/440 (11.13%)	3.01 (2.04–4.43)	**<0.001**	2.48 (1.63–3.76)	**<0.001**
3	Stroke	4583	↑wealth/↓CRP	161/1324 (12.16%)	Reference	–	Reference	–
			↑wealth/↑CRP	75/434 (17.28%)	1.43 (1.09–1.88)	**0.011**	1.37 (1.04–1.81)	**0.027**
			↔wealth/↓CRP	116/1073 (10.81%)	0.89 (0.70–1.13)	0.353	0.87 (0.69–1.11)	0.268
			↔wealth/↑CRP	92/519 (17.73%)	1.46 (1.13–1.89)	**0.004**	1.35 (1.03–1.77)	**0.029**
			↓wealth/↓CRP	101/744 (13.58%)	1.14 (0.89–1.46)	0.288	1.10 (0.85–1.42)	0.489
			↓wealth/↑CRP	102/489 (20.86%)	1.75 (1.36–2.25)	**<0.001**	1.55 (1.18–2.04)	**0.002**
4	Cardiometabolic multimorbidity	3987	↑wealth/↓CRP	23/1190 (1.93%)	Reference	–	Reference	–
			↑wealth/↑CRP	11/389 (2.83%)	1.46 (0.71–3.04)	0.307	0.98 (0.46–2.07)	0.960
			↔wealth/↓CRP	25/940 (2.66%)	1.38 (0.78–2.45)	0.274	1.23 (0.69–2.21)	0.481
			↔wealth/↑CRP	15/454 (3.30%)	1.72 (0.89–3.35)	0.108	1.08 (0.54–2.17)	0.824
			↓wealth/↓CRP	11/628 (1.75%)	0.94 (0.45–1.95)	0.868	0.71 (0.33–1.51)	0.372
			↓wealth/↑CRP	16/386 (4.15%)	2.22 (1.16–4.28)	**0.017**	1.14 (0.55–2.34)	0.726

N.B. effect estimate for models 1–3 is hazard ratio; model 4 is odds ratio.

CHD = coronary heart disease; C.I. = Confidence Interval; CRP=C- reactive protein.

a Age, sex, cohabitation, ethnicity, smoking, physical activity, body mass index, and hypertension.

In terms of our secondary analyses, models to predict incident cardiometabolic multimorbidity (Table 2, Model 4) using logistic regression analyses, revealed those in the lowest wealth tertile and with elevated CRP had over double the risk of developing cardiometabolic multimorbidity over time relative to those in the highest wealth/low CRP group (OR 2.22, 95% C.I. 1.16; 4.28), adjusting for age and sex. Fully adjusted models were non-significant. The age and sex adjusted RERI for cardiometabolic multimorbidity was 0.82 (95% C.I. −1.11; 2.75) suggestive of a non-significant super-additive effect. Additional secondary analyses examined the multiplicative effect of CRP on the relationship between wealth and incident diabetes/high blood glucose, CHD and stroke. Fully adjusted models found no significant multiplicative interaction effect for any of the disease outcomes (all *p* > 0.05).

## 4 Discussion

The results from our study indicate that low SEP and elevated CRP may directly affect cardiometabolic disease, with the combination of both these factors leading to the poorest health outcomes over time. We observed that those in the lowest wealth tertile with elevated CRP were at increased risk of developing CHD, stroke, and diabetes/high blood glucose over 14 years of follow-up compared to those in the highest wealth tertile with low CRP. Our results were also robust to sensitivity analyses removing cases of incident disease within two years of baseline. Moreover, this increased risk extended to incident cardiometabolic multimorbidity. However, this association was not supportive of a significant interaction, suggesting low wealth and elevated CRP do not have a synergistic effect on cardiometabolic disease onset.

Our findings are in line with earlier work to suggest a link between income and cardiovascular (de Mestral & Stringhini, 2017; Marshall et al., 2015) and metabolic (Robbins et al., 2005) health, including both disease onset and prognosis. In addition, research has investigated the link between SEP and mortality in cardiometabolic comorbid patients, finding an inverse association between CVD-related mortality and SEP in those with type 1 diabetes (Rawshani et al., 2015), such that low SEP was associated with greater mortality risk. Moreover, since our sample consisted of adults aged 50 years and older, our findings indicate that inequalities in health persist into older age. Previous work has suggested socioeconomic disparities in health peak in middle and early old age (House et al., 2005), including in relation to income (Mosquera et al., 2016).

Mechanistic studies have indicated low SEP to be associated with the pathogenesis of CHD through its impact on the severity of coronary artery calcification (Steptoe et al., 2010). Such evidence is in line with the hypothesis that low SEP acts as a chronic stressor, capable of causing long-term damage to the hypothalamic-pituitary adrenal (HPA) axis and its negative feedback system. The HPA axis is bidirectionally linked to the immune system, and cortisol is known to have immunomodulatory effects on inflammation in response to stress (DeSantis et al., 2012; Knight et al., 2021). In turn, inflammatory processes are known to be involved in the pathogenesis of atherosclerosis and to be predictive of future cardiac events (Biasucci et al., 2017; Golia et al., 2014). Our work lends supports for this model, by providing evidence for the detrimental effect of having both low wealth and inflammation (CRP). However, we were unable to find evidence of a significant interaction (additive or multiplicative) effect, suggesting the effect of wealth on future health risk is not dependent on CRP levels, but rather both low wealth and elevated CRP each independently carry additional risk for future cardiometabolic disease. Indeed, our findings for models predicting incident stroke revealed that elevated CRP, as opposed to low wealth, were key. While there is some evidence for the role of SEP for predicting stroke (Cho et al., 2019), CRP has a well-established link with stroke (The Emerging Risk Factor Collaboration, 2010). CRP may affect stroke risk via its effects on other markers of inflammation, particularly fibrinogen (The Emerging Risk Factor Collaboration, 2010). Furthermore, conventional risk factors are also likely to play a role in understanding the link between wealth, CRP, and cardiometabolic health. We adjusted for obesity (BMI), hypertension, physical activity and smoking in our models, but other factors such mental wellbeing (Panagi et al., 2021) are also important predictors of future disease onset. We performed sensitivity analyses to include depression in our models, with results upheld.

Our findings provide evidence in favour of the inclusion of CRP in screening measures for cardiometabolic risk. SEP is included under guidelines by the UK National Institute for Health and Clinical Excellence (NICE, 2014). However, the QRISK2 score (ClinRisk Ltd.), also recommended in these guidelines for assessing cardiovascular risk, does not include CRP in its predictive model. Uncertainty around the additional predictive value of CRP in improving such risk models has been subject to debate (Hingorani et al., 2012; Johns et al., 2018). Our findings suggest the need for further work to study how best to stratify patients who stand to benefit most from prophylactic pharmacotherapy and lifestyle modifications, with greater consideration given to the intersecting risk between the social environment and disease biomarkers.

Our work has a number of strengths, including the use of a 14-year follow-up period allowing us to conduct prospective analyses using nationally representative data of middle aged and older adults with control for sociodemographic, lifestyle and biological covariates. We must however acknowledge the limitations of our work. Blood samples were not collected for a large number (∼35%) of participants in ELSA leading to missing CRP data. Due to the small event rate of incident cardiometabolic multimorbidity in our sample, we were unable to investigate temporality in the development of multimorbidity. In addition, our illness outcomes were also self-reported which may introduce measurement error to our results; though there is evidence of the reliability of self-reported physical illness in epidemiological cohort studies (Simpson et al., 2004). Mortality data was not available so competing risk of death has not been taken into consideration in models. Statins have been shown to have anti-inflammatory effects (Antonopoulos et al., 2012); however, we were unable to control for baseline medication use as this data is not available in wave 2 of ELSA. The categorisation of wealth into tertiles and CRP into a binary variable creates 6 groups, making it hard to disentangle the stepwise increase in risk attributable to belonging to one of the four middle groups (i.e. high wealth/high CRP, intermediate wealth/low CRP, intermediate wealth/high CRP, low wealth/low CRP). Creating binary variables out of continuous data (such as wealth and CRP) can reduce statistical power, can underestimate the variation in the outcome variable between categories, and can mask non-linear relationships (Altman & Royston, 2006). Lastly, we did not consider alternative measures of SEP in our analysis such as employment or education.

In conclusion, we have reported evidence for the detrimental effect of both wealth and CRP for predicting future CHD, stroke and diabetes/high blood glucose, and cardiometabolic multimorbidity. Our findings reflect the accumulation of disease risk in which the social determinants of health in combination with elevated inflammation influence disease onset over time.

## Funding sources

RAH is supported by the Academy of Medical Sciences/the Wellcome Trust/the Government Department of Business, Energy and Industrial Strategy/the British Heart Foundation/Diabetes UK Springboard Award [SBF006\1036].

## Declaration of competing interest

All authors report no conflicts of interests.

## Ethical statement

Information on the ethical approval received for each wave of ELSA can be found below:

**ELSA Wave 10** received ethical approval from the South Central – Berkshire Research Ethics Committee on 22nd March 2021 (21/SC/0030).

**ELSA Wave 9** received ethical approval from the South Central – Berkshire Research Ethics Committee on 10th May 2018(17/SC/0588).

**ELSA Wave 8** received ethical approval from the South Central – Berkshire Research Ethics Committee on 23rd September 2015 (15/SC/0526).

**ELSA Wave 7** received ethical approval from the NRES Committee South Central - Berkshire on 28th November 2013 (13/SC/0532).

**ELSA Wave 6** received ethical approval from the NRES Committee South Central - Berkshire on 28th November 2012 (11/SC/0374).

**ELSA Wave 5** received ethical approval from the Berkshire Research Ethics Committee on 21st December 2009 (09/H0505/124).

**ELSA Wave 4** received ethical approval from the National Hospital for Neurology and Neurosurgery & Institute of Neurology Joint Research Ethics Committee on 12th October 2007 (07/H0716/48).

**ELSA Wave 3** received ethical approval from the London Multi-Centre Research Ethics Committee on 27th October 2005 (05/MRE02/63).

**ELSA Wave 2** received ethical approval from the London Multi-Centre Research Ethics Committee on 12th August 2004 (MREC/04/2/006).

## Author statement

**Lydia Poole:** Conceptualization, Methodology, Data curation, Formal analysis, Writing - Original draft preparation, Project administration. **Antonio Lazzarino:** Conceptualization, Writing - Original draft preparation. **Kimberley Smith:** Conceptualization, Writing - Reviewing and Editing. **Ruth Hackett:** Formal analysis, Writing - Original draft preparation, Writing - Reviewing and Editing.

## Data Availability

ELSA data is archived in the UK Data Service
