# Supplementary material for: The combined effect of socioeconomic position and C-reactive protein for predicting incident cardiometabolic disease: Findings from a 14-year follow-up study of the English Longitudinal Study of Ageing (ELSA)
Source: SSM Popul Health. 2023 Sep 25;24:101520. doi: 10.1016/j.ssmph.2023.101520 (PMC10550841; doi:10.1016/j.ssmph.2023.101520)
Supplement: Multimedia component 1 [file mmc1.docx]

**Supplementary Files**

**Table 1. Prospective associations between baseline wealth/CRP additive interaction and incident disease over 14 years, additionally adjusted for baseline glycated haemoglobin**

| Model | Incident disease outcome | N |  | Age and sex adjusted hazard ration (95% C.I.) | *p* | Fully adjusted hazard ratio (95% C.I.) | *p* |
| --- | --- | --- | --- | --- | --- | --- | --- |
| 1 | Diabetes/high blood glucose | 4291 | ↑wealth/↓CRP  ↑wealth/↑CRP  ↔wealth/↓CRP  ↔wealth/↑CRP  ↓wealth/↓CRP  ↓wealth/↑CRP | Reference  2.52 (1.74- 3.63)  1.70 (1.23- 2.35)  3.13 (2.22-4.41)  2.26 (1.60-3.19)  4.47 (3.21-6.24) | -  <**0.001**  **0.001**  **<0.001**  **<0.001**  **<0.001** | Reference  1.54 (1.06-2.25)  1.34 (0.96-1.86)  1.87 (1.31-2.67)  1.72 (1.21-2.45)  1.77 (1.21-2.57) | -  **0.025**  0.087  **<0.001**  **0.003**  **0.003** |
| 2 | CHD | 4102 | ↑wealth/↓CRP  ↑wealth/↑CRP  ↔wealth/↓CRP  ↔wealth/↑CRP  ↓wealth/↓CRP  ↓wealth/↑CRP | Reference  2.12 (1.39-3.23)  1.67 (1.17-2.39)  1.77 (1.15-2.72)  1.98 (1.33-2.93)  3.09 (2.09-4.56) | -  **<0.001**  **0.005**  **0.010**  **<0.001**  **<0.001** | Reference  1.94 (1.26-2.97)  1.63 (1.14-2.33)  1.56 (1.00-2.43)  1.78 (1.19-2.67)  2.49 (1.63-3.79) | -  **0.003**  **0.008**  **0.048**  **0.005**  **<0.001** |
| 3 | Stroke | 4484 | ↑wealth/↓CRP  ↑wealth/↑CRP  ↔wealth/↓CRP  ↔wealth/↑CRP  ↓wealth/↓CRP  ↓wealth/↑CRP | Reference  1.44 (1.09-1.89)  0.88 (0.69-1.13)  1.48 (1.14-1.92)  1.13 (0.88-1.46)  1.73 (1.34-2.23) | -  **0.010**  0.320  **0.003**  0.344  **<0.001** | Reference  1.38 (1.04-1.83)  0.86 (0.68-1.10)  1.37 (1.05-1.79)  1.08 (0.83-1.41)  1.52 (1.15-2.02) | -  **0.027**  0.238  **0.023**  0.549  **0.003** |

^a^ age, sex, cohabitation, ethnicity, smoking, physical activity, body mass index, hypertension and glycated haemoglobin

C.I. = Confidence Interval; CRP = C-reactive protein

**Table 2. Prospective associations between baseline wealth/CRP additive interaction and incident disease over 14 years, additionally adjusted for baseline total cholesterol**

| Model | Incident disease outcome | N |  | Age and sex adjusted hazard ration (95% C.I.) | *p* | Fully adjusted hazard ratio (95% C.I.) | *p* |
| --- | --- | --- | --- | --- | --- | --- | --- |
| 1 | Diabetes/high blood glucose | 4382 | ↑wealth/↓CRP  ↑wealth/↑CRP  ↔wealth/↓CRP  ↔wealth/↑CRP  ↓wealth/↓CRP  ↓wealth/↑CRP | Reference  2.46 (1.71- 3.55)  1.70 (1.24- 2.34)  3.06 (2.18-4.30)  2.31 (1.65-3.22)  4.45 (3.21-6.18) | -  <**0.001**  **0.001**  **<0.001**  **<0.001**  **<0.001** | Reference  1.68 (1.16-2.44)  1.54 (1.12-2.12)  1.83 (1.28-2.61)  1.69 (1.19-2.38)  2.14 (1.49-3.08) | -  **0.007**  **0.008**  **<0.001**  **0.001**  **<0.001** |
| 2 | CHD | 4189 | ↑wealth/↓CRP  ↑wealth/↑CRP  ↔wealth/↓CRP  ↔wealth/↑CRP  ↓wealth/↓CRP  ↓wealth/↑CRP | Reference  2.09 (1.37-3.18)  1.67 (1.18-2.38)  1.75 (1.14-2.69)  1.93 (1.30-2.85)  3.01 (2.04-4.44) | -  **<0.001**  **0.004**  **0.011**  **0.001**  **<0.001** | Reference  1.87 (1.22-2.87)  1.62 (1.14-2.32)  1.54 (0.99-2.40)  1.73 (1.16-2.59)  2.49 (1.64-3.79) | -  **0.004**  **0.008**  0.055  **0.008**  **<0.001** |
| 3 | Stroke | 4582 | ↑wealth/↓CRP  ↑wealth/↑CRP  ↔wealth/↓CRP  ↔wealth/↑CRP  ↓wealth/↓CRP  ↓wealth/↑CRP | Reference  1.43 (1.09-1.88)  0.89 (0.70-1.13)  1.46 (1.13-1.90)  1.14 (0.89-1.46)  1.75 (1.37-2.25) | -  **0.011**  0.355  **0.004**  0.298  **<0.001** | Reference  1.36 (1.03-1.81)  0.87 (0.69-1.11)  1.35 (1.03-1.76)  1.10 (0.85-1.42)  1.56 (1.18-2.05) | -  **0.030**  0.269  0.030  0.477  **0.002** |

^a^ age, sex, cohabitation, ethnicity, smoking, physical activity, body mass index, hypertension and total cholesterol

C.I. = Confidence Interval; CRP = C-reactive protein

**Table 3. Prospective associations between baseline wealth/CRP additive interaction and incident disease over 14 years, excluding cases within two years of baseline**

| Model | Incident disease outcome | N |  | Age and sex adjusted hazard ratio (95% C.I.) | *p* | Fully adjusted hazard ratio (95% C.I.) | *p* |
| --- | --- | --- | --- | --- | --- | --- | --- |
| 1 | Diabetes/ high blood glucose | 3477 | ↑wealth/↓CRP  ↑wealth/↑CRP  ↔wealth/↓CRP  ↔wealth/↑CRP  ↓wealth/↓CRP  ↓wealth/↑CRP | Reference  2.56 (1.70- 3.86)  1.98 (1.40- 2.81)  3.37 (2.31-4.93)  2.17(1.47-3.21)  3.94 (2.67-5.80) | -  **<0.001**  **<0.001**  **<0.001**  **<0.001**  **<0.001** | Reference  1.71 (1.12-2.59)  1.78 (1.25-2.53)  1.99 (1.34-2.96)  1.60 (1.07-2.39)  1.87 (1.22-2.86) | -  **0.013**  **0.001**  **<0.001**  **0.021**  **0.004** |
| 2 | CHD | 3326 | ↑wealth/↓CRP  ↑wealth/↑CRP  ↔wealth/↓CRP  ↔wealth/↑CRP  ↓wealth/↓CRP  ↓wealth/↑CRP | Reference  2.25 (1.41-3.59)  1.65 (1.11-2.47)  1.70 (1.04-2.79)  1.96 (1.26-3.07)  3.22 (2.08-4.98) | -  **<0.001**  **0.015**  **0.036**  **0.003**  **<0.001** | Reference  2.00 (1.24-3.22)  1.59 (1.06-2.38)  1.45 (0.87-2.41)  1.74 (1.10-2.76)  2.56 (1.60-4.10) | -  **0.004**  **0.026**  0.154  **0.018**  **<0.001** |
| 3 | Stroke | 3230 | ↑wealth/↓CRP  ↑wealth/↑CRP  ↔wealth/↓CRP  ↔wealth/↑CRP  ↓wealth/↓CRP  ↓wealth/↑CRP | Reference  2.09 (1.14-3.83)  0.97 (0.54-1.73)  1.53 (0.80-2.92)  1.44 (0.79-2.62)  2.58 (1.44-4.61) | -  **0.018**  0.918  0.201  0.232  **0.001** | Reference  1.84 (0.99-3.43)  0.89 (0.49-1.59)  1.28 (0.65-2.51)  1.22 (0.66-2.25)  1.88 (0.99-3.58) | -  0.055  0.684  0.476  0.532  0.055 |

^a^ age, sex, cohabitation, ethnicity, smoking, physical activity, body mass index, and hypertension

CHD = coronary heart disease; C.I. = Confidence Interval; CRP = C-reactive protein

**Table 4. Prospective associations between baseline wealth/CRP additive interaction and incident disease over 14 years, additionally adjusted for baseline depressive symptoms**

| Model | Incident disease outcome | N |  | Age and sex adjusted hazard ratio (95% C.I.) | *p* | Fully adjusted hazard ratio (95% C.I.) | *p* |
| --- | --- | --- | --- | --- | --- | --- | --- |
| 1 | Diabetes/ high blood glucose | 4342 | ↑wealth/↓CRP  ↑wealth/↑CRP  ↔wealth/↓CRP  ↔wealth/↑CRP  ↓wealth/↓CRP  ↓wealth/↑CRP | Reference  2.48 (1.72- 3.57)  1.69 (1.23- 2.33)  3.00 (2.13-4.22)  2.31 (1.65-3.23)  4.41 (3.18-6.13) | -  <**0.001**  **0.001**  **<0.001**  **<0.001**  **<0.001** | Reference  1.69 (1.17-2.46)  1.52 (1.11-2.10)  1.81 (1.27-2.58)  1.66 (1.18-2.35)  2.12 (1.48-3.05) | -  **0.006**  **0.010**  **0.001**  **0.004**  **<0.001** |
| 2 | CHD | 4152 | ↑wealth/↓CRP  ↑wealth/↑CRP  ↔wealth/↓CRP  ↔wealth/↑CRP  ↓wealth/↓CRP  ↓wealth/↑CRP | Reference  2.11 (1.39- 3.22)  1.66 (1.17- 2.37)  1.74 (1.13-2.68)  1.89 (1.27-2.80)  2.83 (1.91- 4.20) | -  **<0.0010.005**  **0.011**  **0.002**  **<0.001** | Reference  1.96 (1.28-3.01)  1.62 (1.13-2.31)  1.56 (1.00-2.43)  1.68 (1.12-2.53)  2.36 (1.54-3.61) | -  **0.002**  **0.008**  **0.048**  **0.012**  **<0.001** |
| 3 | Stroke | 4543 | ↑wealth/↓CRP  ↑wealth/↑CRP  ↔wealth/↓CRP  ↔wealth/↑CRP  ↓wealth/↓CRP  ↓wealth/↑CRP | Reference  1.44 (1.09- 1.89)  0.90 (0.71- 1.14)  1.46 (1.12-1.89)  1.16 (0.90-1.48)  1.77 (1.38- 2.27) | -  **0.010**  0.391  **0.004**  0.258  **<0.001** | Reference  1.38 (1.04-1.83)  0.88 (0.69-1.11)  1.34 (1.02-1.76)  1.08 (0.83-1.39)  1.52 (1.16-2.01) | -  **0.024**  0.280  **0.034**  **0.581**  **0.003** |

^a^ age, sex, cohabitation, ethnicity, smoking, physical activity, body mass index, hypertension, and depressive symptoms

CHD = coronary heart disease; C.I. = Confidence Interval; CRP = C-reactive protein
